# Multiple model species selection for transcriptomics analysis of non-model organisms

**DOI:** 10.1186/s12859-018-2278-z

**Published:** 2018-08-13

**Authors:** Tun-Wen Pai, Kuan-Hung Li, Cing-Han Yang, Chin-Hwa Hu, Han-Jia Lin, Wen-Der Wang, Yet-Ran Chen

**Affiliations:** 1 Department of Computer Science and Engineering, National Taiwan Ocean University, Keelung, Taiwan; 2 Department of Computer Science and Information Engineering, National Taipei University of Technology, Taipei, Taiwan; 3 Department of Bioscience and Biotechnology, National Taiwan Ocean University, Keelung, Taiwan; 4 Department of Bioagricultural Science, National Chiayi University, Chiayi, Taiwan; 5 Agricultural Biotechnology Research Center, Academia Sinica, Taipei, Taiwan

**Keywords:** RNA-seq, Reference model species, Differential expression analysis, Ultra-conserved orthologous gene, Gene ontology, Biological pathway

## Abstract

**Background:**

Transcriptomic sequencing (RNA-seq) related applications allow for rapid explorations due to their high-throughput and relatively fast experimental capabilities, providing unprecedented progress in gene functional annotation, gene regulation analysis, and environmental factor verification. However, with increasing amounts of sequenced reads and reference model species, the selection of appropriate reference species for gene annotation has become a new challenge.

**Methods:**

We proposed a novel approach for finding the most effective reference model species through taxonomic associations and ultra-conserved orthologous (UCO) gene comparisons among species. An online system for multiple species selection (MSS) for RNA-seq differential expression analysis was developed, and comprehensive genomic annotations from 291 reference model eukaryotic species were retrieved from the RefSeq, KEGG, and UniProt databases.

**Results:**

Using the proposed MSS pipeline, gene ontology and biological pathway enrichment analysis can be efficiently achieved, especially in the case of transcriptomic analysis of non-model organisms. The results showed that the proposed method solved problems related to limitations in annotation information and provided a roughly twenty-fold reduction in computational time, resulting in more accurate results than those of traditional approaches of using a single model reference species or the large non-redundant reference database.

**Conclusions:**

Selection of appropriate reference model species helps to reduce missing annotation information, allowing for more comprehensive results than those obtained with a single model reference species. In addition, adequate model species selection reduces the computational time significantly while retaining the same order of accuracy. The proposed system indeed provides superior performance by selecting appropriate multiple species for transcriptomic analysis compared to traditional approaches.

**Electronic supplementary material:**

The online version of this article (10.1186/s12859-018-2278-z) contains supplementary material, which is available to authorized users.

## Background

High-throughput RNA sequencing (RNA-seq) involves the sequencing of cDNA samples using next-generation sequencing instruments, thus permitting the analysis of complete transcriptomes from samples subjected to different experimental conditions. In addition to providing a snapshot of an organism’s transcriptional profile, RNA-seq can also be used to observe changes in gene expression levels by comparing transcriptomes obtained at various time points or from different tissues under different environmental settings [1]. In recent years, owing to advances allowing for high-throughput, relatively fast, and low-cost experiments, RNA-seq has led to unprecedented development of studies focusing on gene functions and regulation, as well as correlations between genes and environmental factors. Compared to traditional microarray hybridization approaches for gene expression analysis, a major advantage of RNA-seq is that it allows for analysis of non-model target species without comprehensive gene annotations in advance [2]. This is because RNA-seq can still accurately detect novel gene transcripts and expression levels in different tissues or under different conditions through de novo assembly of the sequenced reads. In addition, RNA-seq provides high dynamic expression ranges, and costs are relatively low for obtaining whole-transcriptome data [3]. Based on these advantages, non-model organisms now have been frequently selected by the research community for extensive study. It is also due to the facts that non-model organisms possess many interesting and unique features compared to model organisms, and comprehensive transcriptome analysis reveals novel genes and helps to address genetic evolution and ecological issues from different aspects.

Although transcriptional data can be obtained using RNA-seq without any prior knowledge of gene sequences, for functional genomic analysis of non-model species, a sequence similarity alignment must be performed with reference sequence datasets containing known gene functions in order to accurately annotate the gene functions of assembled sequences. The most popular gene sequence database is the International Nucleotide Sequence Database Collaboration (INSDC), which regularly updates sequence information from three databases: the National Center for Biotechnology Information (NCBI), the European Bioinformatics Institute (EMBL-EBI), and the DNA Data Bank of Japan (DDBJ). The non-redundant (*nr*) sequence database GenBank, maintained by NCBI, contains a popular reference dataset for sequence annotation; thus, several RNA-seq studies performed on novel species have thus far aligned sequences to the *nr* sequence database for gene function annotation [4–6]. However, the volume of this database is enormous. As of June 2016, a total of 37,382,402 sequences had been indexed. Using this dataset as a reference for assembled sequence alignment and gene function annotation not only requires a large amount of computational resources, but the alignment results may also contain false positive annotations due to contaminated samples and false assembled contigs. An alternative method commonly used by biologists is to select the single reference model species that has the greatest evolutionary relevance to the target species. While this method is rapid and simple, the selected model species may not necessarily have complete gene annotations, and thus it is possible that certain information may be missed. Therefore, in this study, we aimed to develop a tool that can use multiple model species simultaneously as references for annotating genes from new RNA-seq data. Selecting only a few reference species possessing complete annotations and close evolutionary relationships with the target species not only circumvents the searching of an entire database but also provides improved gene annotation information when compared with that acquired using a single selected model species, thereby improving the accuracy of any subsequent functional analyses.

In order to select suitable model species, we first integrated the NCBI Taxonomy database from INSDC, which describes phylogenetic relationships among species. This information is used to conduct a phylogenetic analysis between the target species and model species. In addition to the relationship provided by the phylogenetic tree, we further used ultra-conserved orthologs (UCOs) for rapid and accurate screening of model species. These UCOs are a set of 357 single-copy genes originating from *Arabidopsis thaliana* [7]. These proteins are known as UCOs because they are highly conserved among most eukaryotes. In previous experiments, UCO sequences were mostly used to verify the quality of assembled sequences by determining the percentage of UCO genes in the assembled sequences, where a higher percentage would indicate higher quality and a more complete assembly [8–11]. In this study, UCO genes were used to select reference species by identifying similarities between the UCO sequences of the target and model species via sequence alignment, with higher conserved sequence similarities implying closer phylogenetic relationships.

Finally, differentially expressed gene sets were functionally annotated through gene ontology (GO) and biological pathway enrichment analysis based on multiple species selection. GO is a structural vocabulary system defined by the Gene Ontology Consortium, and it provides a standard vocabulary set for gene function annotation [12]. Biological pathway annotation is another method used to analyze the associated functions of gene clusters. Each biological pathway includes a series of interactions among molecules within a cell that might produce certain products or lead to a change in function. Different types of biological pathways are presented for describing different levels of physical and/or regulatory interactions, such as metabolism, gene regulation, and signal transmission pathways [13]. There are more than a hundred pathway databases available for biologists to analyze and model associated biological systems quantitatively. Here, we will only focus on KEGG Pathway database since it is one of the most well-known and comprehensive pathway databases for biologists [14], By using both GO and KEGG pathway analysis for gene function annotations, enrichment analyses of differentially expressed gene clusters of several experiments will be performed and compared to traditional approaches for demonstrating effective and efficient performance of our proposed mechanism.

## Methods

### Dataset collection

For effective and efficient gene functional analysis in this study, a total of 291 eukaryotic reference model species were verified and selected from the Kyoto Encyclopedia of Genes and Genomes (KEGG) [14], Universal Protein Resource (UniProt) [15], and NCBI RefSeq [16] databases simultaneously. These 291 selected species possess comprehensive gene annotations for functional enrichment analysis. The NCBI RefSeq database contains non-redundant, well-annotated, and curated information for genomic DNA, RNA, and protein sequences, and genomes in RefSeq are copies of selected assembled genomes available in GenBank. This annotated information from RefSeq can be accessed through the BLAST, Entrez, and NCBI FTP sites. In order to utilize functional information from the biological pathway and GO annotations, initial integration with both the KEGG and UniProt databases was performed in advance. We collected all annotated genes and corresponding pathway information for differential expression analysis, in particular for the 291 selected model species. In other words, we collected corresponding GO annotations from UniProt and functional molecular interaction relationships from KEGG. The UniProt database is a freely accessible website that provides comprehensive protein sequences and corresponding high-quality, curated functional annotations. Therefore, we retrieved gene information including the protein ID, gene ID, and corresponding GO annotations for the 291 selected model species from UniProt in advance. The downloaded information from these two databases was integrated by mapping the information to protein IDs and gene IDs indexed by the RefSeq database. We also downloaded the NCBI Taxonomy database, which contains almost all species appearing in the NCBI database and the detailed corresponding positions of these species within the phylogenetic tree. Lineage information for all organisms in this database allows users to select appropriate reference species from among the 291 reference model species. The NCBI Taxonomy database is the standard nomenclature and classification repository for the INSDC, comprising the GenBank, ENA (EMBL), and DDBJ databases [17]. It includes the names and taxonomic lineages of species based on either nucleotide or protein sequences collected in the INSDC databases. The taxonomy database is manually curated by a group of scientists at the NCBI who apply current taxonomic literature to maintain a phylogenetic taxonomy for the source organisms represented in the sequence databases. To date, more than 1,460,000 organisms and relative branch nodes are included in the taxonomy database. Finally, a total of 357 UCO protein sequences from the UC Davis Genome Center were also downloaded as references for supporting model species selection through sequence similarity analysis.

### System flowchart

**Fig. 1:**
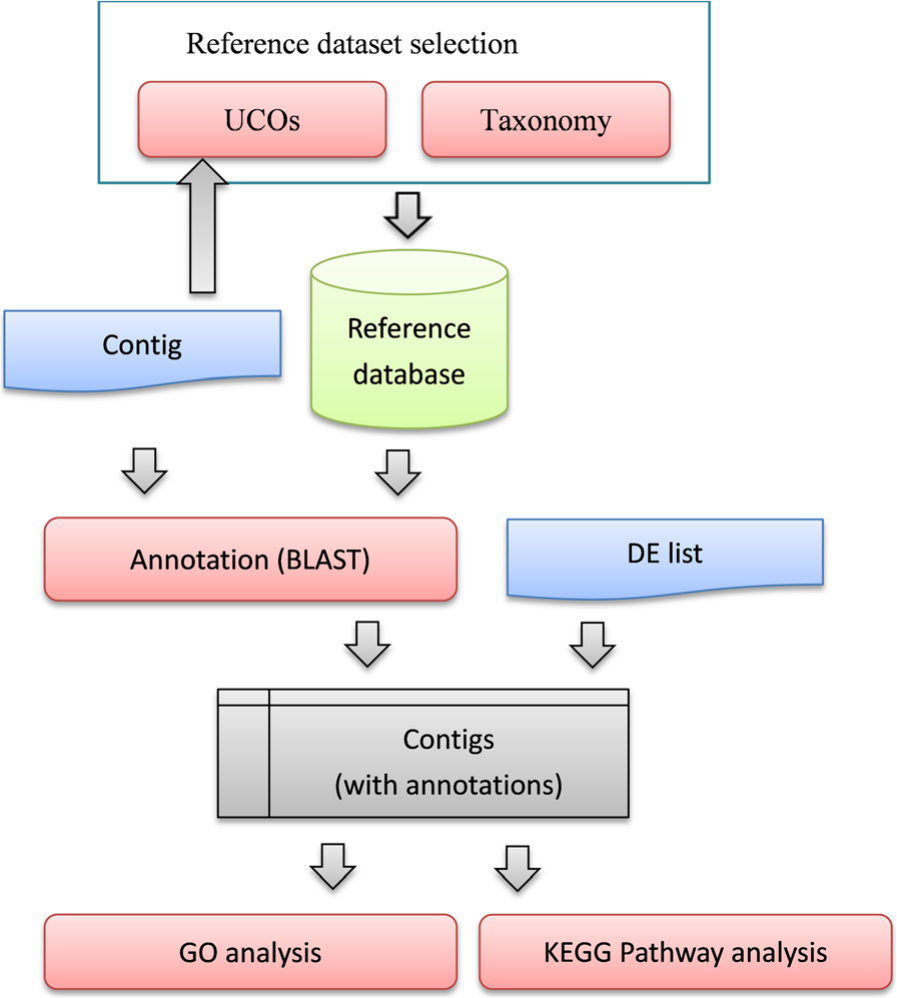
The proposed system configuration. DE represents differential expression

In this study, we proposed a system for differential expression analysis of transcriptomic sequencing datasets. The system intelligently selects appropriate reference species for gene functional annotation and functional enrichment analysis. An overview flowchart of the proposed system is shown in Fig. 1. We postulated that finding appropriate and sufficient reference model species and utilizing comprehensive sequence information from the selected reference model species were crucial to RNA-seq differential expression analysis. The functional module of reference model species selection from among the 291 NCBI RefSeq eukaryotic species candidates can be achieved through two different approaches. The first approach for species selection is achieved by comparing organism lineage relationships as defined by the NCBI Taxonomy. When users input the name of the query species, the proposed system will identify closely related species from the taxonomy tree. An alternative approach performs UCO sequence comparisons among various species. For UCO analysis, only assembled transcriptome contigs in fasta format are required for comparison with collected UCO proteins clustered from the 291 candidate model species (Additional file 1). After UCO sequence comparison, the most similar model species are ranked for later interactive selection. In both cases, protein-coding sequences and annotated information for selected reference model species are then extracted and used to comprise the target database for sequence comparison. Once the model reference species are selected, the assembled contigs are compared against the protein sequences of the selected reference species using the BLAST program. Matched genes with the highest sequence similarity for each query contig are considered as orthologous genes, and related gene functions are annotated for the newly assembled contigs. According to the BLAST results, therefore, gene function annotation is acquired for each assembled contig. For the last step of the proposed pipeline, the system performs functional enrichment analysis using GO and KEGG biological pathway analysis for both annotated contigs and a differentially expressed gene list. The results provide statistically significant biological pathways and GO terms associated with the designed transcriptome sequencing experiments.

### Reference species dataset

For selection of the most appropriate reference model species, two different approaches were proposed. The first approach is based on information in the NCBI Taxonomy tree, and the other is based on similarity analysis of inter-species UCO protein sequence comparisons. The traditional method of selecting reference species has been constrained by the evolutionary relationships among the query and all candidate species. According to the pipeline, if the query species is present in the NCBI Taxonomy database, the system will reveal all possible reference model species with the shortest branch distances to the query species according to the taxonomic tree. However, the query species may not be present in the taxonomy database or it may possess a large number of closely related species located under the same branch node within the taxonomic tree. In order to efficiently select accurate and sufficient reference species, we proposed a novel approach for identifying suitable reference model species by comparing UCOs between the query and all candidate model species.

### Taxonomic tree of candidate species

The NCBI Taxonomy database is the standard nomenclature and classification repository of the INSDC. In the taxonomy tree, there is no clear definition of numeric distances between two nodes. Hence, the evolutionary relationship between any two species can only be represented as their topological locations between nodes on the phylogenetic tree, and accurate evolutionary distances between species pairs cannot be detected from the tree structure. Instead, only relative topological tree structural distances can be used, indicating that a species pair is more related than another species pair with a longer topological tree structural distance. Therefore, in our pipeline, all 291 reference species are compared with the query species to search for their lowest common ancestor (LCA) nodes, and the depths of neighboring nodes are evaluated as a score from the defined taxonomic tree structure. Higher scores represent larger depths from the root and imply that the reference species is located at a relatively lower level compared to that of the query species. The proposed system initially sets a default threshold of three species as a minimum and five as a maximum, but this can be adjusted by the user. The LCA distance between the target species and query species must satisfy that they belong to the same phylum.

### UCO gene set

The UCO gene set contains 357 single-copy genes that represent characteristics that are highly conserved across most eukaryotic organisms. These genes can be found in *A. thaliana*, humans, mice, yeast, fruit flies, *Caenorhabditis elegans*, and other eukaryotic organisms. With their widely conserved characteristics, UCO sequence similarities are used as standards for selecting reference model species. The proposed system suggests that a suitable reference model species should possess more similar gene sequences for improved gene annotations during reference mapping procedures. To achieve this goal, we performed BLAST searches for all 291 model species against the defined UCOs to identify all UCO protein sequences in each model species. The BLASTP program was used with an E-value threshold of less than 1.0E-10. All identified UCOs for each of the 291 model species were saved in the UCO database (Additional file 1). To compare evolutionary distances between the query species and the 291 candidate reference model species, the system uses TBLASTN to identify all possible UCOs from the assembled contigs provided by the user. Once UCO contigs are identified in the query species, the system applies the BLASTX algorithm to search the identified UCO contigs against the representative UCO proteins from the 291 model species. The final UCO mapping results between the query species and the most similar reference species are ranked and recorded for determining reference species suggestions. As a compromise between computational requirements and accuracy, the top three mapped species identified through UCO comparisons are selected as the initial candidate reference model species.

To confirm the suitability of using UCO distance matrices for model species selection, Mantel’s test was applied to verify the correlation between UCO similarity and evolutionary distance between any species pairs. Through multiple species alignment using the Clustal Omega tool, a total of 357 distance matrices for each UCO gene were created. Each matrix is symmetric, and each cell represents the sequence similarity of a specific UCO gene between any two species among the 291 model species. Mantel test is an approach that can verify the correlation between two distance matrices [18]. Different from the correlation coefficient calculation, Mantel test can compare two-dimensional problems. The test is evaluated via permutation procedures, in which the rows and/or columns of the distance matrices are randomly rearranged, and the correlation is determined iteratively to achieve the correlation score between two matrices. A score closer to 1 represents a positive correlation, and a score of 0 indicates that the matrices are completely unrelated. The formula is shown as follows, where *x* and *y* represent two different matrices, *s*_*x*_ and *s*_*y*_ represent standard deviations for *x* and *y,* respectively, and *n* is the dimension of the matrices.

1 $$ \mathrm{r}=\frac{1}{n-1}{\sum}_{i=1}^n{\sum}_{j=1}^n\frac{\left({x}_{ij}-\overline{x}\right)}{s_x}\cdot \frac{\left({y}_{ij}-\overline{y}\right)}{s_y} $$

It should be noted that the total number of mapped UCOs for each species might differ; hence, the dimensions of the two matrices are different. Therefore, for calculating the correlation between two UCO distance matrices, only the species with mapped UCO genes are considered. In other words, only corresponding matrix elements with distance similarities in both distance matrices are calculated. Finally, Mantel test is performed for correlation evaluation of UCO distance matrices. A value close to 1 indicates that use of UCO gene similarities is suitable and consistent for evaluating species relationships and that the UCO gene comparisons are able to suggest reference model species for selection.

### Differential expression analysis

In order to understand the biological meaning of an RNA-seq experiment, KEGG pathway and GO enrichment analysis are performed. Functional enrichment analysis is an approach to analyze and extract important biological themes from the expression information of interactive genes. The associated differentially expressed genes are classified according to previously identified gene annotations, and each KEGG pathway or GO term is analyzed for its calculated probability. In general, there is an expected probability of a certain proportion of genes in a differentially expressed gene cluster being associated with a given KEGG pathway or a GO term. However, if the number of genes involved in this pathway or term is much higher than the expectation, a pathway or a GO term is considered to be significant. In other words, the occurrence of this condition is not due to chance but is inferred to be closely related to the biological experiment. For enrichment analysis, a *P*-value of less than 0.05 according to a hypergeometric distribution analysis is considered to be statistically significant.

## Results and discussion

### Number of model species in MSS system

Based on the outline presented above, we designed a multiple model species selection system, including 291 reference model species. The selection of these species was mainly based on the intersection of the RefSeq, KEGG, and UniProt databases. In addition, all related KEGG pathways and GO term annotations were also collected in the designed integrated database. The collected model species include metazoa, viridiplantae, fungi, and protists. It should be noticed that there is no taxonomic information for protists in the NCBI Taxonomy, and the class of protists is clustered as another branch defined by NCBI. A total of 14 species of protists were included as reference model species.

### Statistics of UCOs for selected model species

All 291 model species were first searched using BLAST for identification of their corresponding 357 UCO genes with an E-value threshold of less than 1E-10. The BLAST results for each UCO against the 291 selected model species were ranked from high to low. All 357 UCOs were found in most of the collected species; however, some species were missing a few UCOs in their reference genome sequences. Among the collected reference model species, more than 84 present of species contained more than 250 UCO genes, and the UCO gene comparisons were considered to be good indicators for reference model species selection for query non-model species analysis. In other words, through UCO gene comparisons, the system suggests appropriate model species that possess close evolutionary relationships with the query species, reducing the probability of selecting inappropriate reference model species due to insufficient genomic sequence content and/or effective gene annotation information.

### Correlation analysis between species relationships and UCOs

**Fig. 2:**
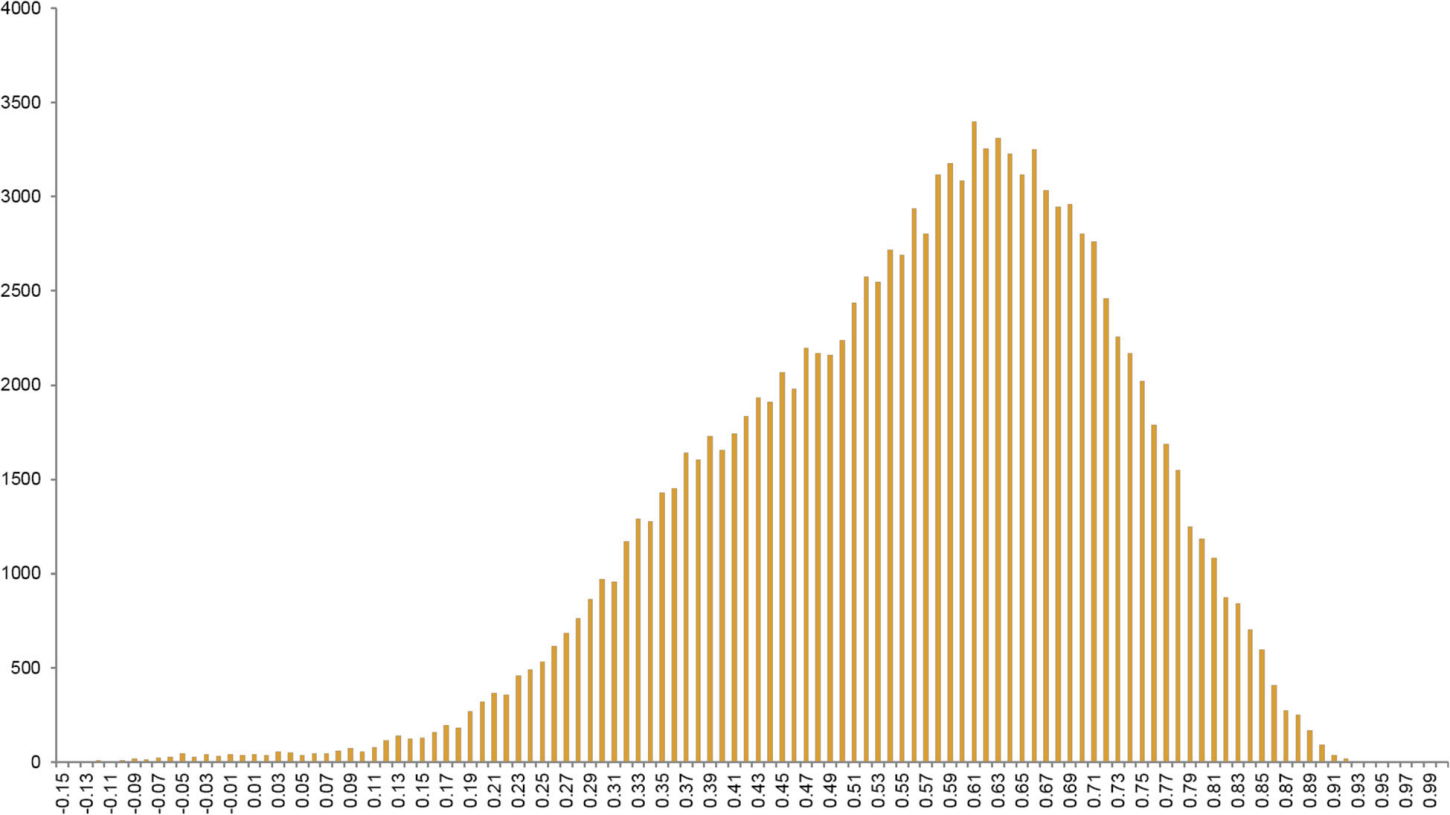
Mantel test correlation coefficients for all possible pairwise combinations of 357 matrices (a total of 63,546 measurements)

To verify the correlation between representative UCOs and phylogenetic relationships among selected species, we performed a pairwise sequence alignment for each representative UCO using all 291 species. The pairwise sequence similarities were calculated and used as evolutionary distances between each pair of species. Hence, a total of 357 matrices were created for each UCO. To evaluate the correlation and consistency among all UCO genes, we performed Mantel test between each pair of created distance matrices. Correlations were evaluated for all possible pairwise combinations of the 357 matrices, creating a total of 63,546 measurements, and the results are shown in Fig. 2. Most correlation scores fall within the range of 0.55 to 0.7. Overall, the average correlation coefficient of 0.56 shows consistency of evolutionary relationships between any two UCO genes. From the correlation analysis, we can see that the representative UCOs from each species demonstrated a high correlation with the evolutionary relationships among species. In other words, when the matched UCO genes from the assembled contigs of a non-model species are used, sequence comparisons with the UCOs from various selected model species accurately reveal phylogenetic relationships in an effective and efficient way. Based on the matched scores, a set of suitable and appropriate reference model species can be confidently suggested for subsequent differential expression analysis.

### Comparison of the UCO & taxonomy approaches

**Fig. 3:**
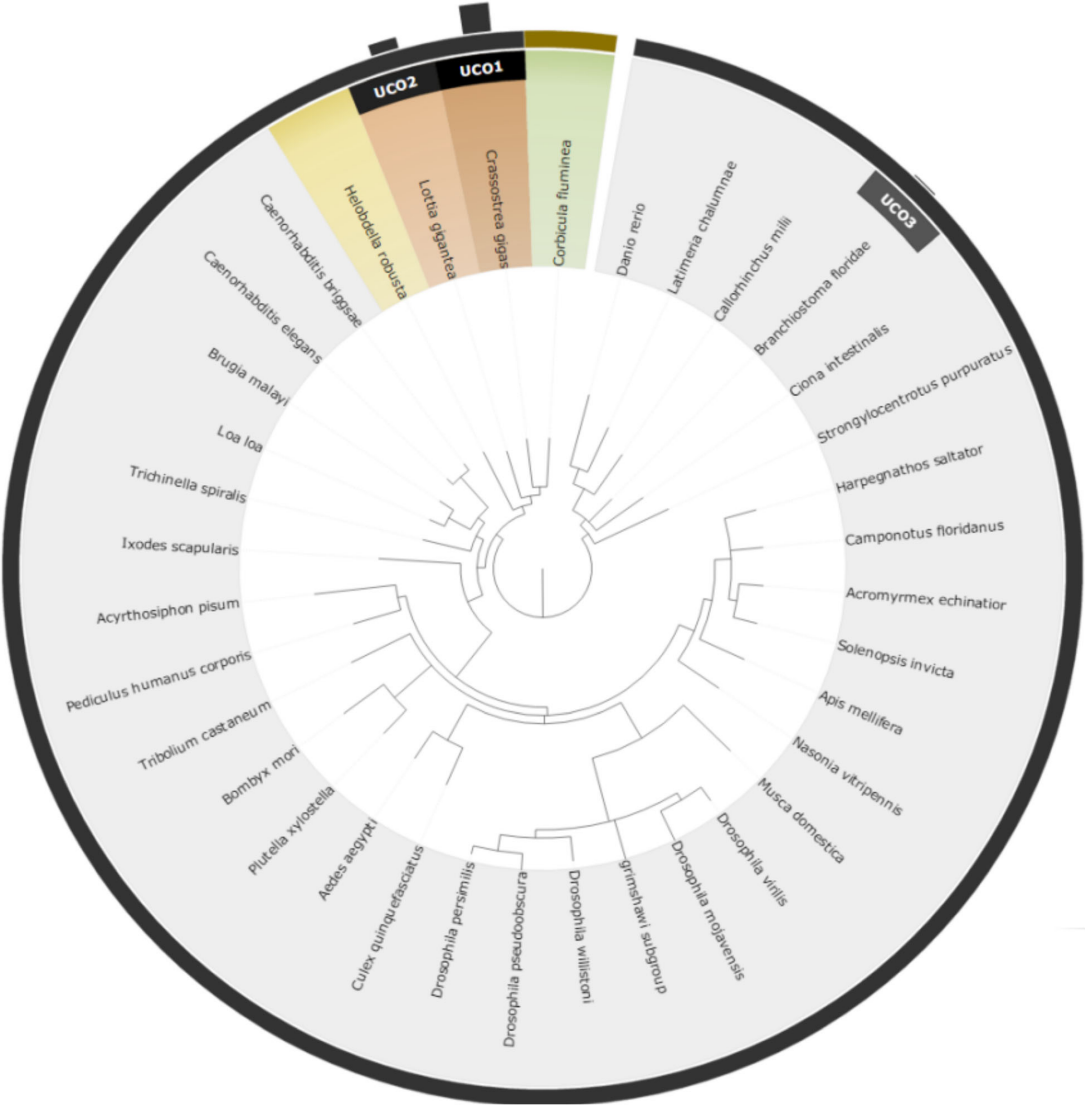
Suggested model species for the non-model species of *Corbicula fluminea* using both taxonomic and UCO approaches. The query species is shown in green. Orange taxa represent selected reference model species using the taxonomic approach, with darker shading indicating closer phylogenetic relationships. Black bars with UCO ranking number indicate the strength of ranking information of suggested reference model species using UCO comparisons

**Fig. 4:**
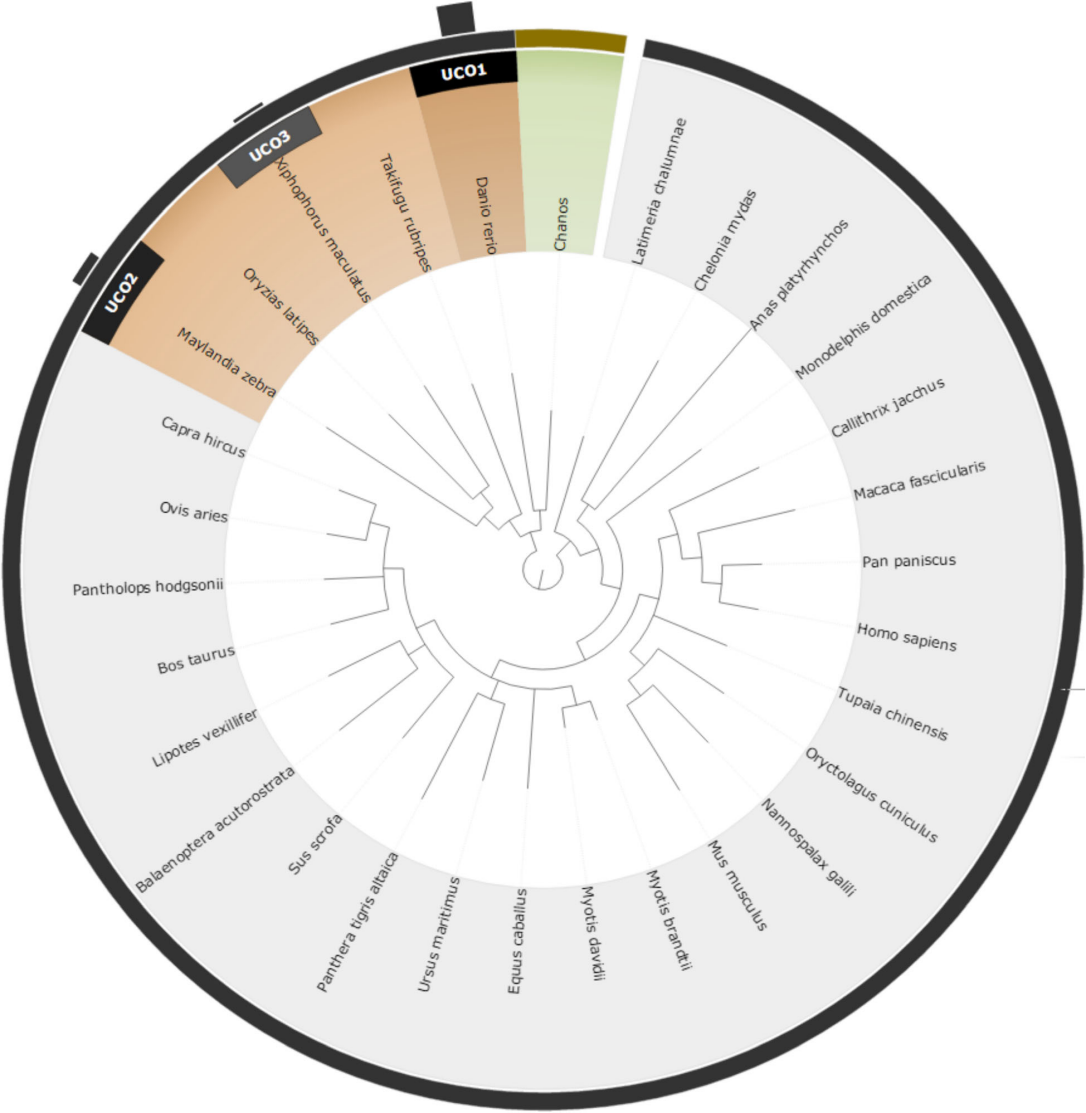
Suggested model species for the non-model species of *Chanos chanos* using both taxonomic and UCO approaches. The query species is shown in green. Orange taxa represent selected reference model species using the taxonomic approach, with darker shading indicating closer phylogenetic relationships. Black bars with UCO ranking number indicate the strength of ranking information of suggested reference model species using UCO comparisons

**Fig. 5:**
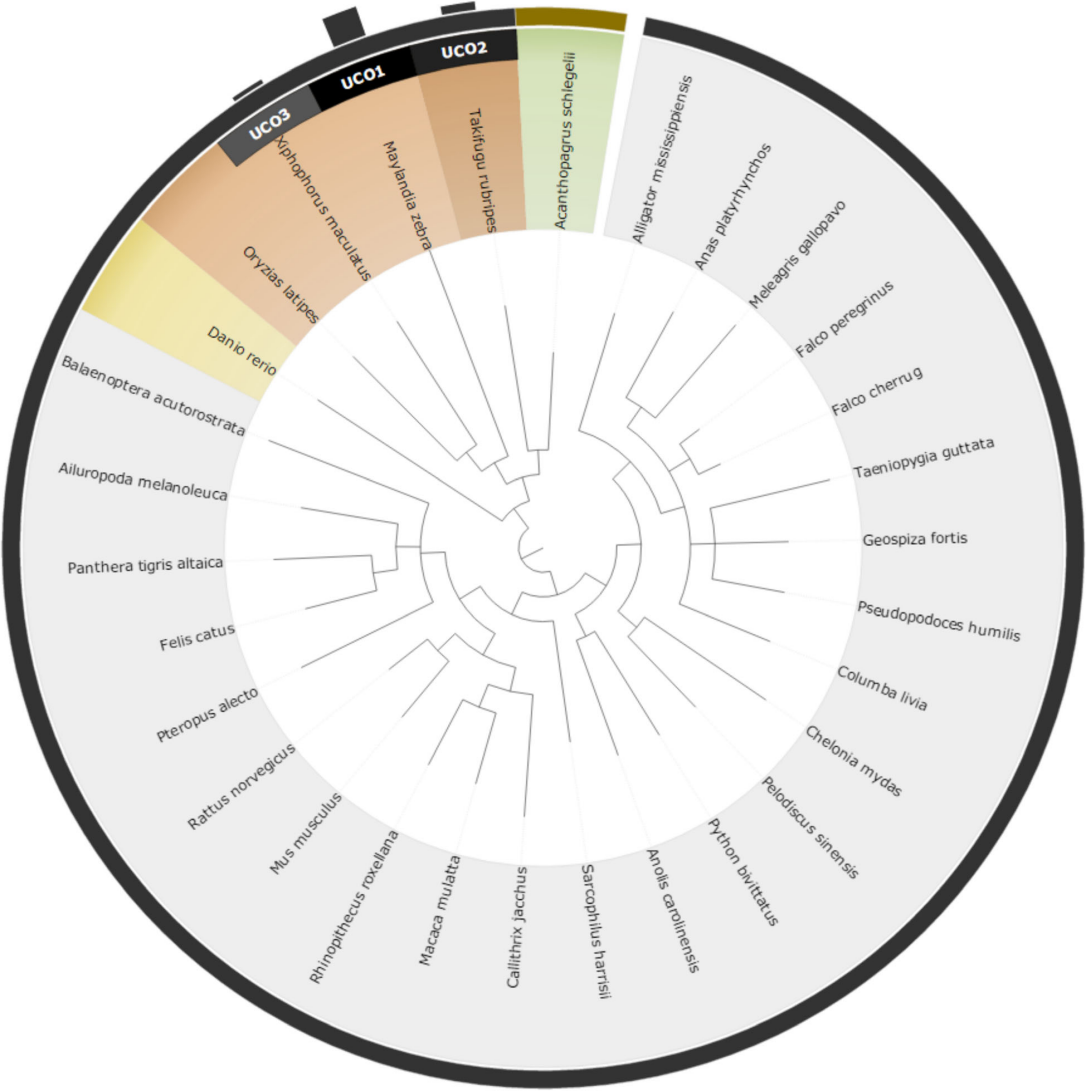
Suggested model species for the non-model species of *Acanthopagrus schlegelii* using both taxonomic and UCO approaches. The query species is shown in green. Orange taxa represent selected reference model species using the taxonomic approach, with darker shading indicating closer phylogenetic relationships. Black bars with UCO ranking number indicate the strength of ranking information of suggested reference model species using UCO comparisons

**Fig. 6:**
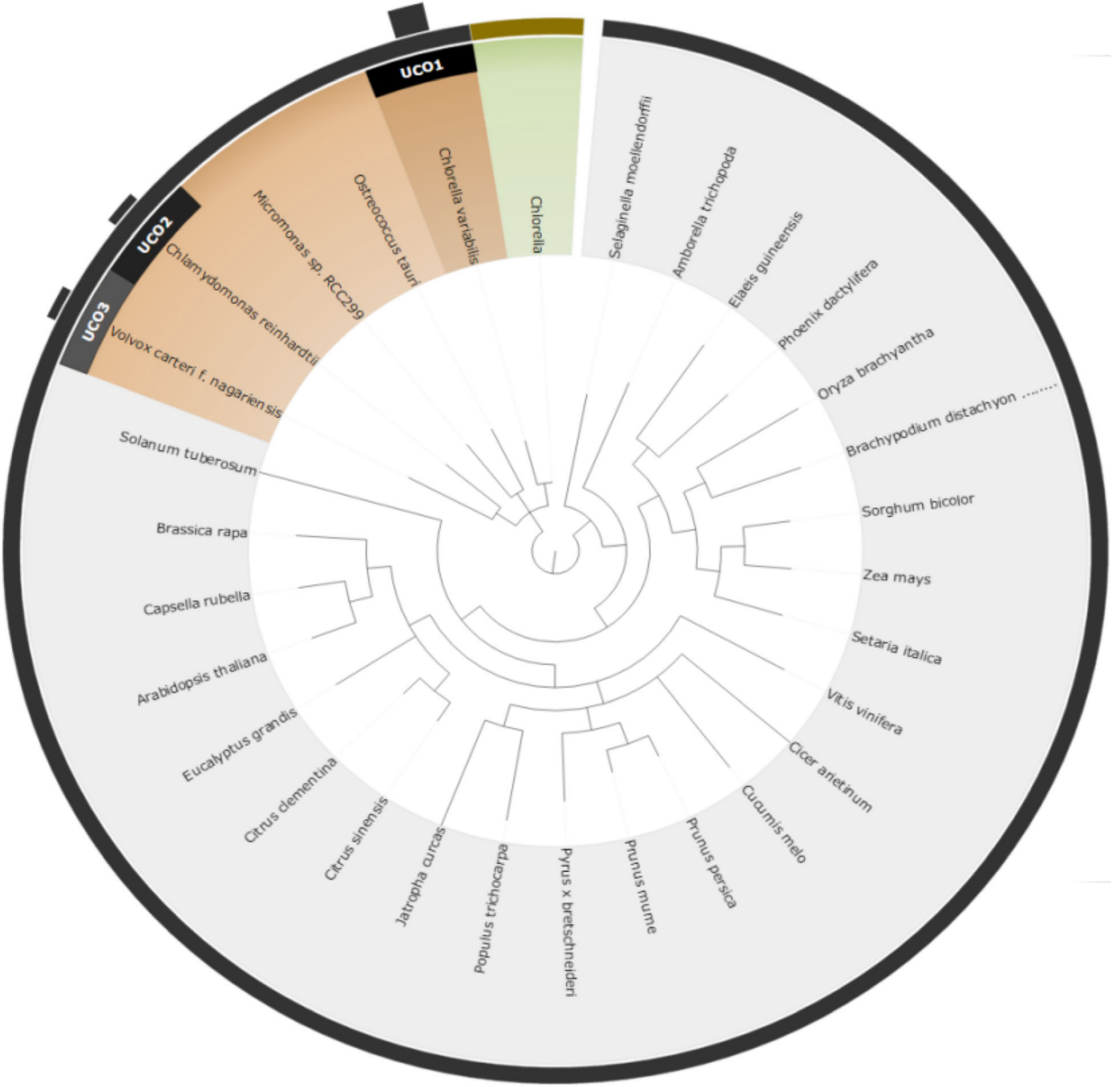
**S**uggested model species for the non-model species of *Chlorella sp.* using both taxonomic and UCO approaches. The query species is shown in green. Orange taxa represent selected reference model species using the taxonomic approach, with darker shading indicating closer phylogenetic relationships. Black bars with UCO ranking number indicate the strength of ranking information of suggested reference model species using UCO comparisons

To compare the two different approaches for reference species selection (UCO and taxonomy approaches), we performed an experimental test using four non-model species: *Corbicula fluminea*, *Chanos chanos*, *Acanthopagrus schlegelii,* and *Chlorella sp.*. The results of the selected species and their corresponding scores are shown in Table 1, while the relationships between the four query species and the recommended reference model species are shown from Figs 3, 4, 5 and 6. Based on the suggested model species, it is clear that the reference model species suggested by the UCO approach overlap with those suggested by the taxonomic approach. However, the ranking orders of the model species differ between the two approaches for the provided examples. Compared to the taxonomic approach, the UCO approach provides a more detailed ranking order when candidate model species possess identical distances from the query based on the coarse taxonomic tree structure. In other words, accurate selection of reference model species and reference mapping procedures for analysis of a non-model species can be achieved. Notably, for the UCO approach, the species name of the query non-model species is not required for RNA-seq analysis. The sequence reads alone provide sufficient information for examining the phylogenetic relationships between the query species and model reference species.

**Table 1:** Selected reference model species using taxonomy-based and UCO approaches for *Corbicula fluminea*, *Chanos chanos*, *Acanthopagrus schlegelii,* and *Chlorella* Sp

Tax ID	Scientific name	Taxonomy		UCO	
45,949	*Corbicula fluminea*	*Crassostrea gigas*	9	*Crassostrea gigas*	231
		*Lottia gigantea*	8	*Lottia gigantea*	85
		*Helobdella robusta*	7	*Branchiostoma floridae*	10
29,144	*Chanos chanos*	*Danio rerio*	21	*Danio rerio*	227
		*Oryzias latipes*	18	*Oryzias latipes*	74
		*Xiphophorus maculatus*	18	*Xiphophorus maculatus*	25
		*Maylandia zebra*	18		
		*Takifugu rubripes*	18		
72,011	*Acanthopagrus schlegelii*	*Takifugu rubripes*	27	*Maylandia zebra*	234
		*Maylandia zebra*	26	*Takifugu rubripes*	62
		*Xiphophorus maculatus*	26	*Xiphophorus maculatus*	32
		*Oryzias latipes*	26		
		*Danio rerio*	19		
3,071	*Chlorella sp.*	*Chlorella variabilis*	9	*Chlorella variabilis*	270
		*Coccomyxa subellipsoidea*	5	*Volvox carteri f. nagariensis*	30
		*Volvox carteri f. nagariensis*	4	*Chlamydomonas reinhardtii*	8
		*Chlamydomonas reinhardtii*	4	*Ostreococcus tauri*	8
		*Ostreococcus tauri*	4		
		*Micromonas sp. RCC299*	4		
		*Micromonas pusilla*	4

### Statistics of reference genes and functional annotations

Next, we showed the statistics of reference genes and corresponding functional annotations according to the selected model species by our proposed system, and these number would be applied for the following differential expression analysis. The RNA-seq datasets of the first experiment were acquired under both normal and hypoxic conditions for the non-model species *Corbicula fluminea*. In this experiment, the first and second reference species suggest by the taxonomic and UCO approaches were identical (*Crassostrea gigas* and *Lottia gigantea*), but the third recommended species was different. The model species *Helobdella robusta* was suggested by the taxonomic approach, while *Branchiostoma floridae* was suggested based on UCO comparisons. Therefore, we performed gene annotation analysis separately using each set of three species recommended by the different approaches. Table 2 shows the total number of CDS sequences retrieved from RefSeq and the number of mapped contigs using BLAST. According to the results of searching the contigs against the suggested reference model species using BLAST, Table 3 shows the statistics for the retrieved GO and KEGG annotations. The results reveal the total number of contigs annotated by GO, the number of associated GO terms, the number of KEGG genes, and the number of associated KEGG pathways. It should be noted that while *Crassostrea gigas* provided the most comprehensive sequence information in the RefSeq database, but the number of GO and KEGG annotations derived from *Crassostrea gigas* are significantly lower than those annotated with the other selected model species.

**Table 2:** The total number of CDS sequences collected from the RefSeq database and mapped contigs of the non-model species *Corbicula fluminea* against different reference species

Scientific name	Number of RefSeq CDS sequences	Number of annotated contigs
*Crassostrea gigas*	45,406	76,787
*Lottia gigantea*	23,822	65,288
*Helobdella robusta*	23,426	45,021
*Branchiostoma floridae*	28,623	58,019

**Table 3:** Annotation statistics for GO terms and KEGG pathways

Scientific name	Contigs annotated by GO	GO term annotations	KEGG genes	KEGG pathways
*Crassostrea gigas*	8223	19,202	4,897	337
*Lottia gigantea*	43,188	99,806	29,471	329
*Helobdella robusta*	32,267	76,051	24,932	334
*Branchiostoma floridae*	42,640	142,310	30,031	339

### Gene ontology analysis

**Fig. 7:**
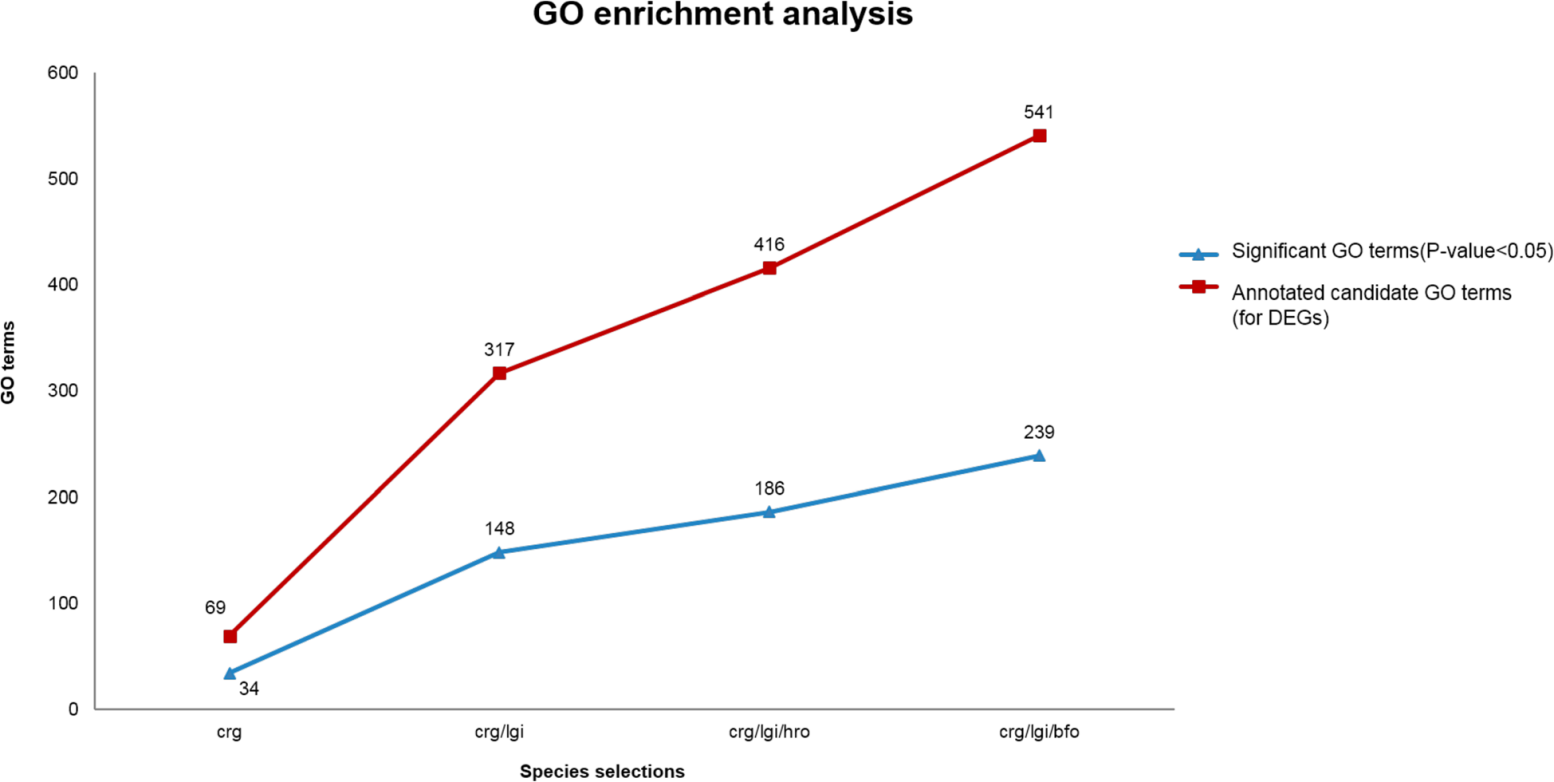
Statistics of identified GO terms following differential expression analysis using different combinations of reference species. DEG, differentially expressed gene; crg, *Crassostrea gigas*; lgi, *Lottia gigantea* (lgi); hro, *H. robusta* (hro); bfo, *B. floridae*

For gene ontology analysis, we performed four different species selections of gene enrichment analyses for comparison: (1) using a single reference species, *Crassostrea gigas* (crg); (2) using the top two species suggested by UCO and taxonomic approaches, *Crassostrea gigas* (crg) and *Lottia gigantea* (lgi); (3) using the top two species plus *H. robusta* (hro), as recommended by the taxonomy-based approach; (4) using the top two selected species plus *B. floridae* (bfo), as recommended by the UCO approach. Figure 7 shows the statistical results of identified GO terms using the four different combinations of reference model species. It can be discovered that when we increase the number of reference model species, the number of associated GO annotations also increases significantly. This indicates that the use of a single reference model species might lead to the loss of important information when the selected species contains incomplete annotations. Hence, increasing the number of relevant model species may solve this problem and compensate for the lack of annotation information compared with the use of a single model reference species.

Based on the identified GO terms obtained from the four different reference model species groups, we specifically investigated GO terms with low *P*-values. Several studies have reported the identification of GO terms associated with hypoxic environments. For example, the chitin metabolic process, calcium ion binding, and Notch signaling pathway have been shown in previous reports to be strongly associated with such conditions [19, 20]. Comparing the results of the third species group (using the three species based on taxonomic classification) with those of the second group (only the top two species included), a total of 38 additional significant GO terms were found, while the fourth experiment (using the three species recommended by the UCO approach) increased the total by an additional 91 significant GO terms. These additional GO terms included single-organism transport, ion transmembrane transporter activity, cytoskeletal protein binding, and hydrolase activity, among which the cytoskeletal protein binding and hydrolase activity functions have been confirmed to be associated with hypoxic environments [21]. In summary, through multiple reference species selection, we were able to use additional transcriptomic information for analysis of differentially expressed genes.

### KEGG pathway analysis

For biological pathway analysis, we performed differential expression analysis using the same four groups of reference species as in the previous section: crg, crg/lgi, crg/lgi/hro, and crg/lgi/bfo. Table 4 shows several significant pathways with *P*-values less than 0.05 for the various groups of model species, with only significant P-values shown. For example, the cell adhesion molecule (CAM) and prion disease pathways were identified only after increasing the number of reference model species, and both of these pathways have been confirmed to be strongly associated with hypoxia in related research reports [22, 23]. A hypoxic environment induced the expression of adhesion molecules in a study of cancer cells, reflecting the fact that hypoxia-induced factors (HIFs) significantly enhance their adhesion to vascular endothelial cells. This enhancement further facilitates hematogenous metastasis of cancers and tumor angiogenesis. Another study of the effect of prion protein accumulation and neuroprotection in mice showed that the prion protein induces protective effects in hypoxic brain damage. In summary, in this case, gene functional analysis based on only a single reference species would have led to missing certain significant pathways owing to the problem of missing annotation information. Hence, by selecting multiple appropriate species for transcriptomic analysis, we were able to improve the accuracy of functional analysis.

**Table 4:** Significant *P*-values according to differential expression analysis of biological pathways

Pathway ID	Pathway name	crg	crg/lgi	crg/lgi/bfo	crg/lgi/hro^a^
ko04514	Cell adhesion molecules (CAMs)				8.03E-05
ko05020	Prion diseases		8.12E-03	8.10E-03	5.95E-04
ko04610	Complement and coagulation cascades		1.14E-03	1.14E-03	1.84E-03
ko04330	Notch signaling pathway		2.51E-03	2.14E-03	4.98E-03
ko00601	Glycosphingolipid biosynthesis - lacto and neolacto series		2.80E-03	2.80E-03	6.23E-03
ko04974	Protein digestion and absorption		2.31E-02	2.30E-02	5.74E-03
ko00740	Riboflavin metabolism	9.46E-03	4.17E-02		
ko04977	Vitamin digestion and absorption				1.04E-02
ko04540	Gap junction		1.06E-02	1.05E-02	2.37E-02
ko04978	Mineral absorption		1.58E-02	1.37E-02	2.49E-02
ko05150	Staphylococcus aureus infection		1.41E-02	1.41E-02	1.79E-02
ko00350	Tyrosine metabolism	6.47E-02	1.58E-02		2.49E-02
ko05130	Pathogenic Escherichia coli infection		2.31E-02	2.05E-02	3.60E-02
ko04512	ECM-receptor interaction	4.66E-02	2.31E-02	2.30E-02	
ko04145	Phagosome		2.74E-02	2.44E-02	
ko00965	Betalain biosynthesis		2.80E-02		
ko04391	Hippo signaling pathway - fly				2.94E-02
ko04210	Apoptosis		4.74E-02	3.74E-02	

^a^crg: *Crassostrea gigas*; lgi: *Lottia gigantean*; hro: *H. robusta*; bfo: *B. floridae*

## Conclusions

This paper proposes a novel approach for efficiently identifying appropriate reference model species by analyzing taxonomic relationships and comparing UCOs. Based on UCO genes largely conserved across all eukaryotic organisms, accurate evolutionary distances are calculated using sequence alignment and applied to compensate for indistinguishable characteristics of the taxonomic tree. In this study, we performed RNA-seq experiments in four non-model species, and our results confirmed that evolutionary distances between species could be ascertained using UCO gene sets. We also performed enrichment analysis of the identified differentially expressed genes using GO and biological pathway approaches. For example, though GO analysis of *Corbicula fluminea* under hypoxic conditions, we identified additional significant GO terms, including the Notch signaling pathway, cytoskeletal protein binding, and hydrolase activity. These GO terms have been found to be associated with hypoxia in reported studies. For biological pathway analysis, additional significant biological pathways were identified, such as the CAM pathway, by increasing the number of multiple species selected as well. Therefore, appropriate selection of multiple species for transcriptomic analysis can reduce required computational time and unnecessary searches against the non-redundant gene dataset. Taking the *Corbicula fluminea* RNA-seq datasets as an example (a total of 393,503 assembled contigs), we applied the non-redundant gene dataset (37,382,402 reference sequences) and the 4 selected model species including *Crassostrea gigas*(45,406 sequences), *Lottia gigantea*(23,822 sequences), *H. robusta*(23,426 sequences), and *B. floridae*(28,623sequences) as our target reference datasets individually. The TBLASTX algorithms were performed under a Linux server with 2 CPU(8 cores), 128GB RAM, and 16 multiple thread settings. The results showed that the proposed method solved problems related to limitations in annotation information and provided a roughly twenty-fold reduction in computational time when selected 3 model species according to taxonomic or UCO approaches. In addition, selecting multiple appropriate species as reference model species helps to reduce missing annotation information, allowing for more comprehensive results than those obtained with a single model reference species. We have developed an integrated system that combines the multiple model species selection pipeline and enrichment analysis of differentially expressed genes. This includes a total of 291 reference model species that were initially selected from the intersection of the RefSeq, KEGG, and UniProt online databases. Corresponding genomes, gene annotations, UCO genes, GO terms, and KEGG pathways were initially constructed and integrated into the proposed system. However, due to different naming architectures among the three databases and an increased number of curated genomes recently, additional model species present in the three databases should be carefully verified and added to the system in the future for a more comprehensive system.

## Additional file

Additional file 1: The identified UCO datasets for 291 candidate reference model species (Additional_UCO_291.fasta). (FASTA 46754 kb)

## Abbreviations

bfo: *B*. *floridae*
crg: *Crassostrea gigas*
DE: Differential Expression
GO: Gene Ontology
hro: *H*. *robusta*
lgi: *Lottia gigantea*
MSS: Multiple species selection
UCO: Ultra-conserved orthologs
